# A ganglion cyst at the elbow causing superficial radial nerve compression: a case report

**DOI:** 10.1186/1752-1947-2-122

**Published:** 2008-04-25

**Authors:** John McFarlane, Ravi Trehan, Miguel Olivera, Carl Jones, Simon Blease, Paul Davey

**Affiliations:** 1 Department of Orthopaedics, Kingston Hospital, Galsworthy Road, Kingston-upon-Thames, Surrey KT2 7QB, UK; 2 Department of Orthopaedics, East Surrey Hospital, Canada Avenue, Redhill, Surrey RH1 5RH, UK

## Abstract

**Introduction:**

We report a rare case of a ganglion cyst at the elbow causing neurological symptoms by stretching the superficial radial nerve alone. Ganglia associated with radial nerve palsy at the elbow have been reported previously involving the deep branch of the posterior interosseous nerve and the superficial radial nerve, but not the superficial radial nerve alone.

**Case presentation:**

A 45-year-old woman presented with a 4-month history of a painful lump in the anterior aspect of her left elbow associated with altered sensation in the dorsoradial aspect of her left hand. There was no history of trauma or any exacerbating factors. On examination the altered sensation was in the superficial radial nerve distribution and she had a positive Tinel's sign over the site of the swelling which was located over the anterior aspect of the radiocapitellar joint.

**Conclusion:**

The unique clinical symptoms and signs of our diagnosis of superficial radial nerve compression were confirmed by magnetic resonance imaging and then operative findings.

## Introduction

The aetiology and pathogenesis of ganglia remain obscure, but degenerative changes at the joint and repeated minor trauma often seem to be a factor in their development. The cysts are usually attached to the underlying adjacent joint capsule, tendon or tendon sheath.

The radial nerve branches into the motor branch (posterior interosseous nerve, PIN) and sensory branch (superficial branch) at the elbow. The superficial branch of the radial nerve is a cutaneous and articular nerve that descends into the forearm under the cover of the brachioradialis and then crosses the roof of the anatomical snuffbox to supply the skin of the dorsum of the hand. Ganglia can occur proximal to the proximal edge of supinator muscle and to the arcade of Frohse or distal to this position. Most reported cases have been located just anterior to the radial head, causing a nerve palsy as the mass pushes the entire radial nerve, including the PIN and the superficial nerve, anteriorly. The arcade of Frohse is a site where the PIN is readily compressed. As the superficial nerve does not pass under the arcade it tends to avoid compression by ganglia [1].

## Case presentation

A 45-year-old woman presented with a 4-month history of a painful lump in the anterior aspect of her left elbow associated with altered sensation in the dorsoradial aspect of her left hand. There was no history of trauma or any exacerbating factors. On examination the altered sensation was in the superficial radial nerve distribution and she had a positive Tinel's sign over the site of the swelling which was located over the anterior aspect of the radiocapitellar joint. There was no weakness in the motor function of the muscles innervated by the PIN.

**Figure 1:**
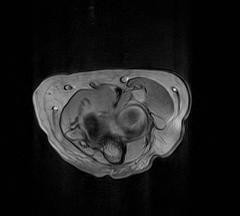
Gradient Echo T2* axial magnetic resonance imaging scan showing a round cystic structure displacing the neurovascular structures in the arcade of Frohse.

**Figure 2:**
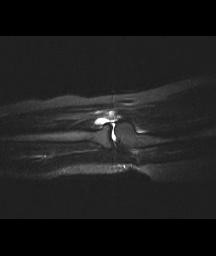
Short T1 inversion recovery fat suppressed sagittal magnetic resonance imaging scan showing a deeper part of the ganglion and its relation to a small effusion in the radiocapitellar joint.

**Figure 3:**
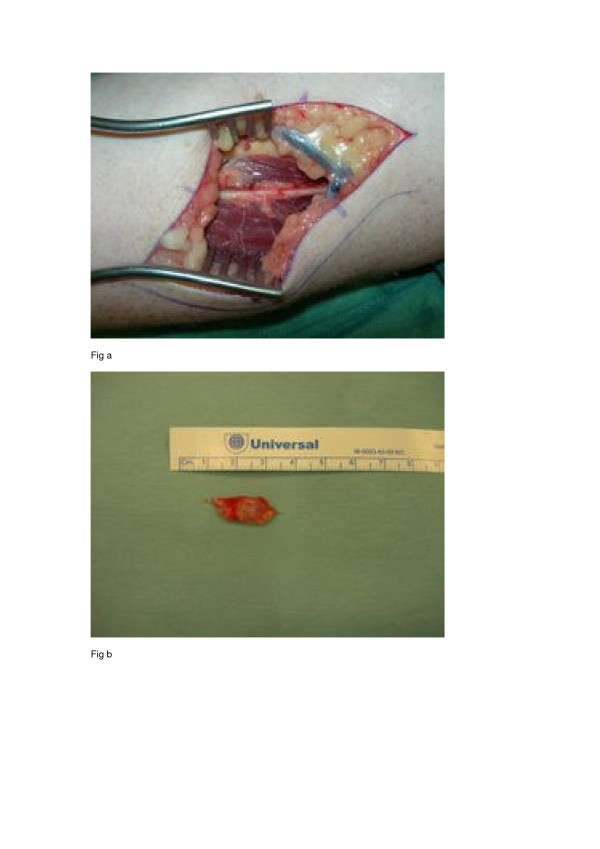
**Figures showing dissection and resection of the ganglion swelling**. (a) Dissection showing the superficial branch of the radial nerve stretched over the ganglion which is located on the anterior aspect of the elbow joint. (b) The ganglion resected in its entirety together with the stalk originating from the radiocapitellar joint.

An ultrasound scan showed an ill-defined cystic lesion measuring approximately 3 cm in length and up to 1.4 cm in depth within the soft tissues on the anterior aspect of the elbow joint, closely related to the anterior aspect of the radius. A magnetic resonance imaging (MRI) scan was carried out and this showed a bilobed cystic mass over the anterior margin of the radiocapitellar joint extending over the anterior surface of the neck of the radius. It was seen to be intimately related to the radial nerve as it passed over the arcade of Frohse and at the division of the PIN. The appearances were typical of a ganglion cyst (Figures 1 and 2). As the patient's symptoms remained severe, exploration of the area was carried out under an axillary block using an anterior approach. Dissection showed that the superficial branch of the radial nerve was stretched over the ganglion which was located on the anterior aspect of the elbow joint (Figure 3a). The ganglion was resected in its entirety together with the stalk originating from the radiocapitellar joint (Figure 3b). Histology of the sample confirmed a multiloculated fibrofatty ganglion cyst with no discernible epithelial lining. The patient was reviewed in the clinic a month later and her symptoms had resolved.

## Discussion

Compression neuropathies that involve the radial nerve in the arm, elbow and forearm are relatively common and can happen anywhere along the course through direct trauma, external compression or a mass.

Most previously reported cases of radial nerve compression have been located just anterior to the radial head, proximal to the arcade of Frohse [2]. Yamazaki reported 14 patients presenting with incomplete paralysis of the extensors of the wrist and fingers due to a ganglion at the elbow, located proximal to the arcade of Frohse in 13 cases and distal in 1, all causing PIN palsy [3]. Matsubara et al. reported eight cases of radial nerve palsy due to ganglions at the elbow proximal to the arcade of Frohse, with compression of the deep and superficial branches seen in three cases, although paraesthesia was noted in only one of the cases, the others presenting with either no symptoms or heaviness of the elbow [4]. Bowen and Stone reported a ganglion in the supinator muscle involving compression of the posterior interosseous nerve causing an extensor weakness in the wrist [5].

## Conclusion

The unique clinical symptoms and signs of our diagnosis of a ganglion causing superficial radial nerve compression were confirmed by MRI and then operative findings.

## Competing interests

The authors declare that they have no competing interests.

## Authors' contributions

JM drafted the manuscript. PD carried out the operation and read and approved the final manuscript. MO assisted with the operation, helped draft the manuscript and reviewed the patient in the clinic. CJ took the photographs and also reviewed the patient in the clinic. RT helped to draft the manuscript and SB carried out and reported on the imaging. All authors read and approved the final manuscript.

## Consent

Written informed consent was obtained from the patient for publication of this case report and any accompanying images. A copy of the written consent is available for review by the Editor-in-Chief of this journal.
